# Comprehensive analysis of human coronavirus antibody responses in ICU and non-ICU COVID-19 patients reveals IgG3 against SARS-CoV-2 spike protein as a key biomarker of disease severity

**DOI:** 10.1099/jmm.0.002012

**Published:** 2025-05-13

**Authors:** Fatma H. Ali, Giusy Gentilcore, Hadeel T. Al-Jighefee, Sara Ahmad Taleb, Ali Ait Hssain, Hamda A. Qotba, Asmaa A. Al Thani, Laith J. Abu Raddad, Gheyath K. Nasrallah, Jean-Charles Grivel, Hadi M. Yassine

**Affiliations:** 1 Biomedical Research Center, QH Health, Qatar University, Doha, Qatar; 2 Department of Biomedical Sciences, College of Health Sciences, QU Health, Qatar University, Doha, Qatar; 3 Deep Phenotyping Core, Sidra Medicine, Doha, Qatar; 4 College of Health and Life Sciences, Hamad Bin Khalifa University, Doha, Qatar; 5 Medical Intensive Care Unit, Hamad Medical Corporation, Doha, Qatar; 6 Primary Health Care Centers, Doha, Qatar; 7 Department of Population Health Sciences, Infectious Disease Epidemiology Group, Weill Cornell Medicine-Qatar, Doha, Qatar

**Keywords:** antibody responses, antigen bead array, biomarker, coronaviruses, COVID-19, cross-reactivity

## Abstract

**Introduction.** Pre-existing immunity to human coronaviruses (HCoVs) may shape the immune response in COVID-19 patients. Increasing evidence suggests that immune cross-reactivity between SARS-CoV-2 and other coronaviruses may determine clinical prognosis.

**Hypothesis.** SARS-CoV-2 disease severity is influenced by pre-existing immunity to HCoVs, with distinct antibody profiles and cross-reactivity patterns.

**Aim.** To investigate the antibody response of ICU and non-ICU SARS-CoV-2 patients against different HCoV proteins and assess the potential impact of pre-existing immunity on SARS-CoV-2 disease outcomes.

**Methodology.** This study used a comprehensive HCoVs antigen bead array to measure antibody response to pathogenic Middle East respiratory syndrome coronavirus (MERS-CoV), SARS-CoV, SARS-CoV-2 and the four seasonal HCoVs in 70 ICU and 63 non-ICU COVID-19 patients.

**Results.** Our analysis demonstrates an overall higher antibody response in ICU than in non-ICU COVID-19 patients. Interestingly, the anti-S1 IgG and IgA were significantly higher among ICU than in non-ICU patients. Similarly, the anti-S1 IgG against NL63 showed a lower response among ICU compared to non-ICU. Cross-reactivity was evident between SARS-CoV-2 and SARS-CoV antibodies but not with MERS-CoV and seasonal HCoVs. The subclass analysis of antibodies recognizing SARS-CoV-2 revealed that anti-S1 IgG1, IgG3, IgA1 and IgA2 were significantly higher in ICU compared to non-ICU. The predominant IgA subtype among SARS-CoV-2 patients was IgA1. We applied machine learning algorithms to subclass serological responses to build classifiers that could distinguish between ICU patients and patients with milder COVID-19. Out of 90 variables used in two different types of models, the variable of highest influence in determining the ICU status was IgG3 against SARS-CoV-2 S, and the top 8 variables of influence included the presence of IgG3 against S-trimer as well as IgA against SARS-CoV-2 S.

**Conclusion.** Understanding the complexities of humoral immunity in various patients is critical for early medical intervention, disease management, selective vaccination and passive immunotherapy.

## Introduction

SARS-CoV-2, the causative agent of COVID-19, is an enveloped positive-sense ssRNA virus. Its genome encodes structural proteins, such as the homotrimer spike (S), envelope (E) and membrane (M) proteins [1]. SARS-CoV-2 belongs to the Betacoronavirus genus and is the seventh coronavirus member known to infect humans [2,3], among which SARS-CoV and Middle East respiratory syndrome coronavirus (MERS-CoV) may also cause lethal diseases in humans. Four additional human coronaviruses (HCoVs) are less pathogenic (HCoV-OC43, HCoV-HKU1, HCoV-NL63 and HCoV-229E), causing mild upper respiratory tract infections, referred to as ‘common cold’.

COVID-19 mainly affects the respiratory system, but other organs may also be involved. The clinical manifestation of COVID-19 ranges from asymptomatic to severe pneumonia, with respiratory failure and even death in up to 5% of cases [4]. In many cases, COVID-19 is asymptomatic, with positive detection of SARS-CoV-2 nucleic acid by RT-PCR, with neither typical clinical symptoms nor apparent lung abnormalities in images [5]. There is increasing evidence that pre-existing immunity against other HCoVs may influence the clinical outcome in SARS-CoV-2-infected patients [6,8].

IgM antibodies are produced around 5–7 days post-SARS-CoV-2 infection [9]. IgG antibodies are usually detected around 11 days and peak at 3–4 weeks post-infection [9]. SARS-CoV-2 antibodies have the shortest detection time (mean of 11 days) in comparison to SARS-CoV-1 (mean of 13.5 days) and MERS-CoV (mean of 15 days) [9]. It has been reported that SARS-CoV-2-specific IgA can be detected after the appearance of IgM and dominates the early neutralizing response [10]. The level of antibodies against SARS-CoV-2 starts to decline within 3 months following a mild and asymptomatic infection [11]. SARS-CoV-2 infection elicits an antibody response even in critically ill patients, regardless of their low circulating lymphocyte counts [12]. IgG and IgA antibody responses differ in their level and durability among mild and critically ill patients [12].

Neutralizing antibodies are suggested as potential correlates of protection against COVID-19 [13]. Many studies illustrated that individuals without previous SARS-CoV-2 infection had varying levels of pre-existing antibodies that could cross-react with SARS-CoV-2 and may influence SARS-CoV-2 immunity [7,14, 15]. Anderson *et al*. [16] found higher levels of anti-OC43 antibodies in pre-pandemic cross-reactive SARS-CoV-2 antibodies [16]. Another study demonstrated that antibodies in healthy individuals were cross-reactive to SARS-CoV-2 and MERS-CoV N proteins [8]. Around 85% of SARS-CoV-2 patients also had reactive antibodies to SARS-CoV-1, whereas 12% were reactive to MERS-CoV [8]. A recent study demonstrated a cross-reactive IgG response in COVID-19 patients against common HCoVs HKU1, OC43, 229E and NL63 [17], while others have shown that antibodies against MERS-CoV and SARS-CoV-1 were cross-reactive with SARS-CoV-2 [18,20].

Betacoronaviruses S proteins share structural homology in multiple regions and could be targets for cross-reactive antibody responses [15]. The S2 subunit is a highly conserved sequence among SARS-CoV-2 and SARS-CoV-1 (90% amino acid sequence identity) [21]. The SARS-CoV-2 S protein shares around~75% of its amino acid sequence with SARS-CoV-1, ~50% with MERS-CoV and 25–30% with seasonal HCoVs S proteins [22,23]. Therefore, pre-existing antibodies against seasonal HCoVs likely target conserved regions of SARS-CoV-2, including the S2 subunit [24]. Another study illustrated that cross-reactive antibodies from pre-pandemic serum samples mediate anti-SARS-CoV-2 ADCC activity [25]. The potential protection conferred by cross-reactive antibodies has been established by inhibiting S-trimer binding to ACE-2 by the serum of MERS-CoV-infected camels [26], supporting the possible protective role of cross-reactive antibodies in SARS-CoV-2 infection, as recently suggested [27,28]. Angiotensin-converting enzyme 2 (ACE2), present on several types of human cells, especially the epithelial cells lining the respiratory tract. It acts as the main entry point for SARS-CoV-2, allowing the virus to infect the body. Identifying cross-reactive antibody epitopes can help guide reasonable vaccination and therapy that target multiple highly pathogenic coronaviruses [15]. In addition to the specificity, the isotypes of antibodies, particularly IgG3, produced in response to SARS-CoV-2 have been shown to play an essential role in the efficiency of viral neutralization *in vivo* [29] and *in vitro* [29], as do dimeric IgA1 [30]. However, the role of antibody isotypes in disease severity has been questioned [31,33].

In this study, we used an in-house flow-cytometric antigen bead array to investigate the magnitude of antibody responses against the SARS-CoV-2 proteins Envelope, Nucleoprotein, Spike-trimer or its fragments S1 and receptor binding domain (RBD), as well as the S1 proteins or the other HCoVs. We used this 11 antigens protein array to measure the total antibody response (IgG, IgM and IgA) and the subclass responses (IgG1, IgG3, IgA1 and IgA2) in 70 ICU and 63 non-ICU COVID-19 patients, along with 22 pre-pandemic serum samples. We quantified the levels and the isotypes of antibodies reactive to viral proteins from HCoVs. We determined whether these antibodies were cross-reactive with SARS-CoV-2 and the association between these antibody responses and disease severity.

## Methods

### Sample collection

In total, 133 serum samples from RT-PCR-confirmed SARS-CoV-2 patients were selected, consisting of ICU (*n*=70) and non-ICU (*n*=63) patients. SARS-CoV-2 ICU samples were collected 1–2 weeks post-infection (first positive PCR) during the patient’s ICU admission. Furthermore, SARS-CoV-2 non-ICU blood and nasal samples were simultaneously collected with those from ICU patients, while only nasal samples were used for diagnosis. Twenty-two pre-pandemic serum samples were collected from healthy blood donors and were used as the negative control group. Disease outcomes were defined using the World Health Organization (WHO) COVID-19 clinical severity scale [34], a nine-point system: uninfected (0=no clinical or virological evidence of infection), ambulatory (1=asymptomatic and 2=activity limitation), mild (3=hospitalized without oxygen supplementation and 4=hospitalized with oxygen supplementation), severe (5=high flow nasal cannula or noninvasive positive pressure ventilation, 6=intubation and invasive mechanical ventilation and 7=mechanical ventilation with additional organ failure support) and 8=death. All non-ICU patients had a score of 1 (asymptomatic). Among the patients, 16 had a score of 8 (death), while 54 had a score between 5 and 8 and were classified as having a severe disease outcome. The clinical characteristics of the patients are shown in Table 1. The samples were presented in 2020–2021 for patients suffering from SARS-CoV-2. These samples represent the first wave of SARS-CoV-2 in Qatar, which was predominantly characterized by the B.1.428 variant, followed by the B.1 variant [35]. Non-ICU samples were collected from a hospital setting or professional laboratory acquisition for routine testing. The samples were collected from asymptomatic patients who tested positive for SARS-CoV-2 via PCR. These samples were collected early in the pandemic before any patients were vaccinated. The age of ICU patients ranged from 30 to 91 years old, with a median of 49 years (interquartile range: IQR=15). In the ICU group, the number of males (*n*=67, 96%) was higher than females (*n*=3, 4%). The age of non-ICU patients ranged from 13 to 61 years old, with a median of 37 years (IQR=20). In the non-ICU group, the number of males (*n*=52, 83%) was higher than that of females (*n*=6, 10%). For five patients, the demographic data weren’t provided. All non-ICU patients were asymptomatic. Data on previous exposure to seasonal HCoVs were unavailable for the patients included in this study. However, a recently published systematic review reported a high prevalence of seasonal HCoVs globally [36]. Among these, HCoV-OC43 was the most prevalent (51.3%), followed by HCoV-NL63 (22.4%), HCoV-HKU1 (7.9%) and HCoV-229E (7.9%). Based on this data, it is assumed that most, if not all, patients have pre-existing immunity against seasonal HCoVs before sample collection. Furthermore, to our knowledge, none of the patients had prior exposure to SARS-CoV-1 or MERS-CoV. Pre-pandemic samples were used as a control to establish a baseline, accounting for any pre-existing antibody responses due to previous exposure.

**Table 1.** The clinical characteristics of patients

Clinical presentation (No. of patients)		ICU (*n*=70)	Non-ICU (*n*=63)
**Age (median, range)**		49 (30–91) years	37 (13–61) years
**WHO clinical severity scale**		Severe disease (5–7 score) 54 patientsDeceased (8 score) 16 patients	Ambulatory (1 score) 63 patients
**Gender**	Males	67 (96%)	52 (82.5%)
	Females	3 (4%)	6 (9.5%)
	Not provided	–	5 (7.9%)
**Vaccination**		70 (100%) Not vaccinated	63 (100%) Not vaccinated

### Flow cytometry analysis

**Fig. 1.**
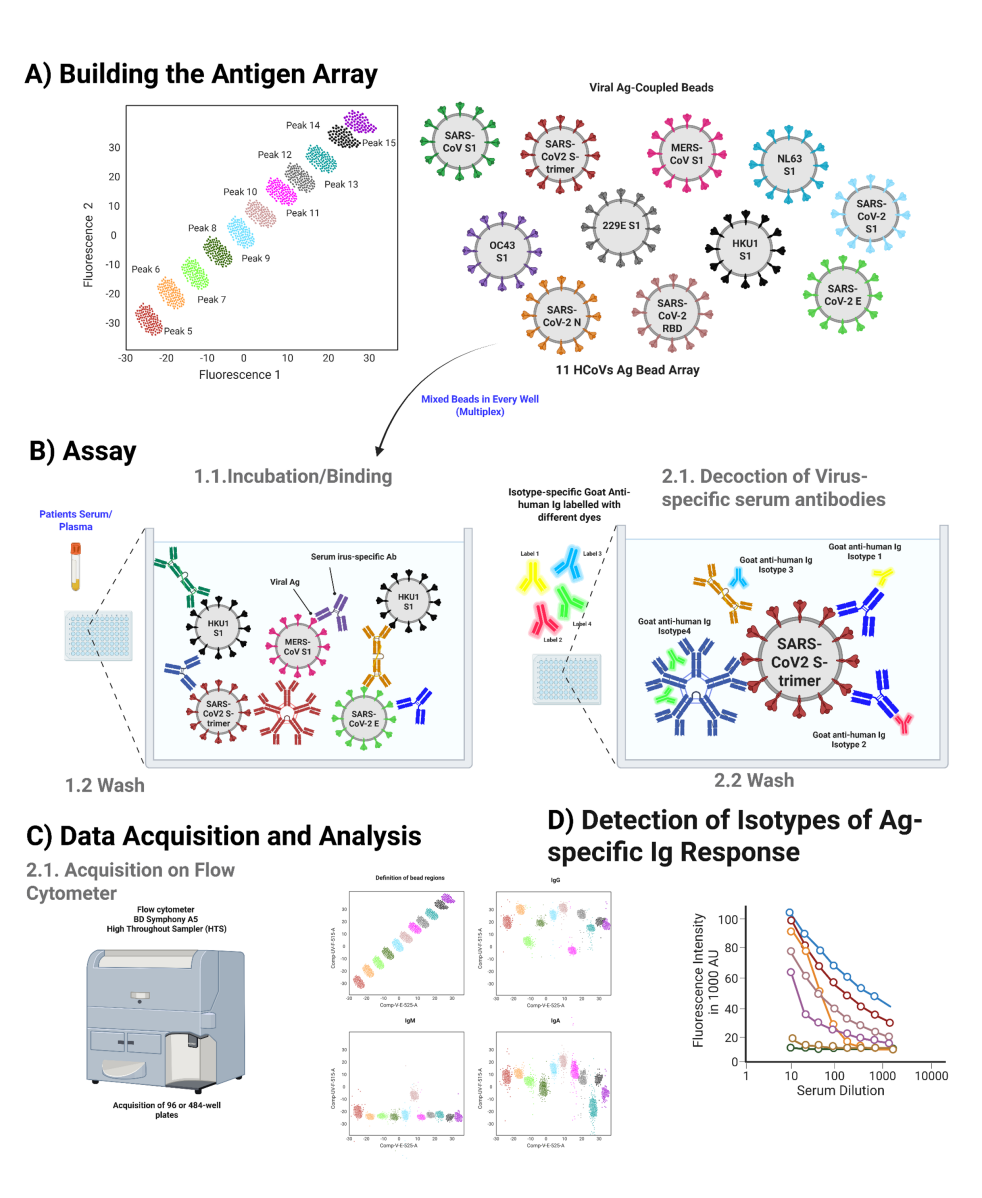
Serological bead array assay. (**a**) Building of the antigen array: 11 carboxymethylated bead sets with distinct fluorescence intensity profiles are coupled with recombinant HCoVs S1 proteins and SARS-CoV-2 spike proteins S-trimer, S1, RBD, Env and Nucleoprotein. (**b**) Assay steps: mixed beads are distributed in filter well plates and incubated with diluted serum samples (step 1.1). The plates are washed (step 1.2) and incubated with a mixture of labelled detection antibodies specific for Ig isotypes or subclasses (step 2.1). The plates are washed, and the beads are resuspended in the assay buffer (step 2.2). (**c**) The plates are acquired on a five-laser BD Symphony A5 using a high-throughput sampler. (**d**) Beads are classified based on their unique fluorescence intensity in two channels, UVF-515 and VE-525. The fluorescence corresponding to each detection antibody is recorded and used to calculate RIs on each bead set.

The presence of antibodies against selected HCoV proteins in the serum is measured using a home-built antigen bead array analysed by flow cytometry (Fig. 1).

### Building of bead array

The bead array was built using a set of 11 carboxymethylated beads with 11 different intensities of UV-excitable dye, providing a unique fluorescence signature for each set (Spherotech) (Fig. 1a). Each bead set was individually coupled to histidine-tagged recombinant HCoVs proteins expressed in human cells (AcroBiosystems, Newark, DE, USA). We included five SARS-CoV-2 proteins or protein fragments derived from the original SARS-CoV-2 Wuhan-Hu-1 strain. The SARS-CoV-2 S1 protein corresponds to the region 16–685 of the S protein and includes a C-terminal His-Tag (AcroBiosystems, catalogue # S1N-C52H4). The SARS-CoV-2 RBD domain corresponds to fragment 319–541 of the S protein, featuring a C-terminal His-Tag (AcroBiosystems, catalogue # SPD-C52H3). The trimeric Spike protein corresponds to the region 16–1,213 of the S protein, incorporating trimer-stabilizing mutations and suppressing the furin cleavage site F817P, A889P, A942P, K986P, V987P, R683A and R685A. It also has a C-terminal His-Tag (AcroBiosystems, catalogue # SPD-C52H3). We also included Envelope protein His-Tag (ThermoFisher, catalogue # RP-87682) and Nucleoprotein His-Tag (AcroBiosystems, catalogue # NUN-C5227). We also included the S1 proteins of MERS His-Tag (Sino Biological, catalogue # 40069-V08H), HCoV-229E His-Tag (AcroBiosystems, catalogue # SIN-V52H4), HCoV-HKU1 His-Tag (AcroBiosystems, catalogue # SIN-V52H6), HCoV-NL63 His-Tag (AcroBiosystems, catalogue # SIN-V52H3) and HCoV-OC43 His-Tag (Sino Biological, catalogue # 40607-V08H1). Therefore, the array consisted of 11 antigens, including 5 SARS-CoV-2 antigens and 6 HCoV S1 proteins. Coupling was performed according to the procedure published previously [37] with a slight modification consisting of the buffer exchange of reconstituted lyophilized recombinant proteins with PBS pH 7.4 using Zeba columns (Pierce). Briefly, 15×106 microspheres were washed in diH2O and activated by resuspension in 100 mM monobasic sodium phosphate, pH 6.2, 5 mg ml^−1^ sulfo-NHS (Pierce, catalogue # 24520) and 5 mg ml^−1^ EDC (Pierce, catalogue # 77149) under shaking for 20 min at room temperature. The activated microspheres were then washed three times with PBS, pH 7.4. Pelleted washed activated beads were resuspended in 1 ml of PBS pH 7.4 containing 100 µg of recombinant proteins and incubated overnight at room temperature under rotation. Coupled microspheres were washed twice with PBS-TBN (0.2% Tween-20, 0.1% BSA, 0.05% sodium azide), resuspended in a final volume of 1 ml, counted using a volumetric-acquisition controlled flow cytometer (Cytek Aurora) and stored at 4 °C in PBS-TBN until further use. Viral antigen coupling was confirmed by performing a binding titration of a His-Tag-specific phycoerythrin (PE) labelled monoclonal antibody (BioLegend, catalogue # 362603).

### Serological assay

#### Measurement of total IgM, IgG and IgA anti-HCoVs antigens

Serum samples diluted 1:20 in assay buffer (10 mM Tris-HCl, pH 7.5, 0.1% BSA, 0.01% Tween-20) were incubated with the bead array (2,000 microspheres for each peak) in a total volume of 50 µl in a Multiscreen HV filter plate (Millipore, catalogue # MSHVN4510) under shaking at 800 r.p.m. for 35 min at room temperature. After three vacuum washes with assay buffer using a PALL vacuum manifold (PALL). The microspheres were incubated with 50 µl of assay buffer containing 0.6 µg ml^−1^ of AlexaFluor 488-labelled goat anti-human IgG polyclonal antibodies (SouthernBiotech, catalogue # 2040–30), 0.63 µg ml^−1^ PE-labelled goat anti-human IgA polyclonal antibodies (Jackson ImmunoResearch, catalogue # 109-115-011) and 1.2 µg ml^−1^ AlexaFluor 647-labelled goat anti-human IgM polyclonal antibodies (SouthernBiotech, catalogue # 2020–31) for 20 min at room temperature under agitation at 800 r.p.m. The microspheres were then vacuum-washed three times in wash buffer (10 mM Tris-HCl, pH 7.5, 0.05% Tween-20) (Fig. 1b), resuspended in the same buffer, acquired and analysed on a BD FACS Symphony A5 equipped with UV (355 nm), Violet (450 nm), Blue (488 nm), YellowGreen (561 nm) and Red (633 nm) lasers and a high-throughput sampler (Fig. 1c).

#### Measurement of IgG and IgA subclasses

Serum samples diluted 1:20 in assay buffer (10 mM Tris-HCl, pH 7.5, 0.1% BSA, 0.01% Tween-20) were incubated with the bead array (2,000 microspheres for each peak) in a total volume of 50 µl in two Multiscreen HV filter plates (Millipore, catalogue # MSHVN4510) under shaking at 800 r.p.m. for 35 min at room temperature. After three vacuum washes with assay buffer using a PALL vacuum manifold (PALL), the microspheres in one plate were incubated with 50 µl of assay buffer containing 1 µg ml^−1^ of AlexaFluor 488-labelled mouse monoclonal antibody 4E3 anti-IgG1 hinge (SouthernBiotech, catalogue # 9052–30), 1 µg ml^−1^ of PE-labelled mouse monoclonal antibody HP605 anti-IgG3 hinge (SouthernBiotech, catalogue # 9210–09) and 1 µg ml^−1^ mouse monoclonal antibody HP602 anti-IgG2 Fc (SouthernBiotech, catalogue # 9710–31) for 20 min at room temperature under agitation at 800 r.p.m. The microspheres in the other plate were incubated with 50 µl of assay buffer containing 1 µg ml^−1^ of PE-labelled mouse monoclonal antibody B3506B4 anti-IgA1 Fc (SouthernBiotech, catalogue # 9130–30) and 1 µg ml^−1^ of APC-labelled mouse monoclonal antibody A96042 anti-IgA2 Fc (SouthernBiotech, catalogue # 9140–31). Following the incubation, the plates were then vacuum-washed three times in wash buffer (10 mM Tris-HCl, pH 7.5, 0.05% Tween-20), resuspended in the same buffer, acquired and analysed as described for total IgG, IgA and IgM measurement.

### Data acquisition and analysis

Bead classification was performed by defining 11 gates in bivariate plots of UV 515 (UV excitation, emission 515/30) fluorescence versus Violet 525 (405 excitations, emission 525/50) fluorescence. Each gated bead population was analysed for the presence of IgM, IgG and IgA, revealed by the fluorescence intensity in B-520 for AlexaFluor488 (488 nm excitation, 525/50 emission), YG-586 form PE (561 nm excitation, 586/14 emission) and R670 for AlexaFluor647 (640 excitations, 670/30 emission) (Fig. 1d). The data were analysed using FlowJo software. An average of 300 beads per region was acquired. The mean fluorescence intensity (MFI) of each bead peak in the fluorescent channels listed above was used in subsequent calculations.

### Response index

The positivity of tested samples was measured by determining a positivity index for each antigen and antibody type. The response index (RI) (Equation 2) is defined as the ratio of the patient’s response MFI to the MFI response of a pooled negative control for the same antigen/isotype. Pooling negative samples dilutes any potential immune response from previous encounters with seasonal coronaviruses. The calculation of RI is illustrated by Equations 1 and 2 below:

MFI of pooled negative controls=MFI of negative controls+3 SD Equation 1

RI=MFI subject/MFI of pooled negative control Equation 2

A response is considered positive when the RI≥1.

### Statistical analysis

Statistical analysis was performed using GraphPad Prism software version 10.0 (GraphPad Software, La Jolla, CA, USA). Data are plotted as scatter plots showing the mean value and sem intervals on a log10 scale. Significant differences between ICU and non-ICU patients were determined using an unpaired t-test adjusted for multiple comparisons by the Holm–Šidák correction [38,39]. Statistical significance was determined when the corrected value *P* was inferior to 0.05. Significance levels were assigned as *P≤0.05, **P≤0.01 and ***P≤0.001.

### Machine learning

Machine learning was performed using the R ‘Classification And REgression Training’ package. This R package provides a standardized interface for working with different machine learning algorithms, allowing an easy switch between different classifiers. It implements various cross-validation strategies to generate more stable estimates through the trainControl function, such as *k*-fold cross-validation and repeated cross-validation. It implements stratified sampling for imbalanced datasets and relies on automated parameter tuning to optimize the finding of hyperparameters through adaptive resampling. This package is used to build class prediction models [40] and is a well-established package in machine learning [41,42]. The dataset was split, with 80% of the data generating a training set and 20% a test set.

Total IgM, IgG and IgA (bulk responses) and IgG1, IgG2, IgG3, IgA1 and IgA2 in response against the SARS-CoV-2 antigens (Nucleoprotein, Envelope, S-trimer, S1 and RBD), as well as the patient’s age, a total of 41 variables were used to generate the models. Models were trained by repeated cross-validation with 5 K folds repeated 10 times. We used the random forest (RF) models and deduced the variables of importance report. We also used the gradient-boosted machine (GBM) models with Bernoulli function loss to report the variable of influence in classifying the ICU patients.

## Results

### Antibody responses to SARS-CoV-2 antigens are higher in ICU patients

**Fig. 2.**
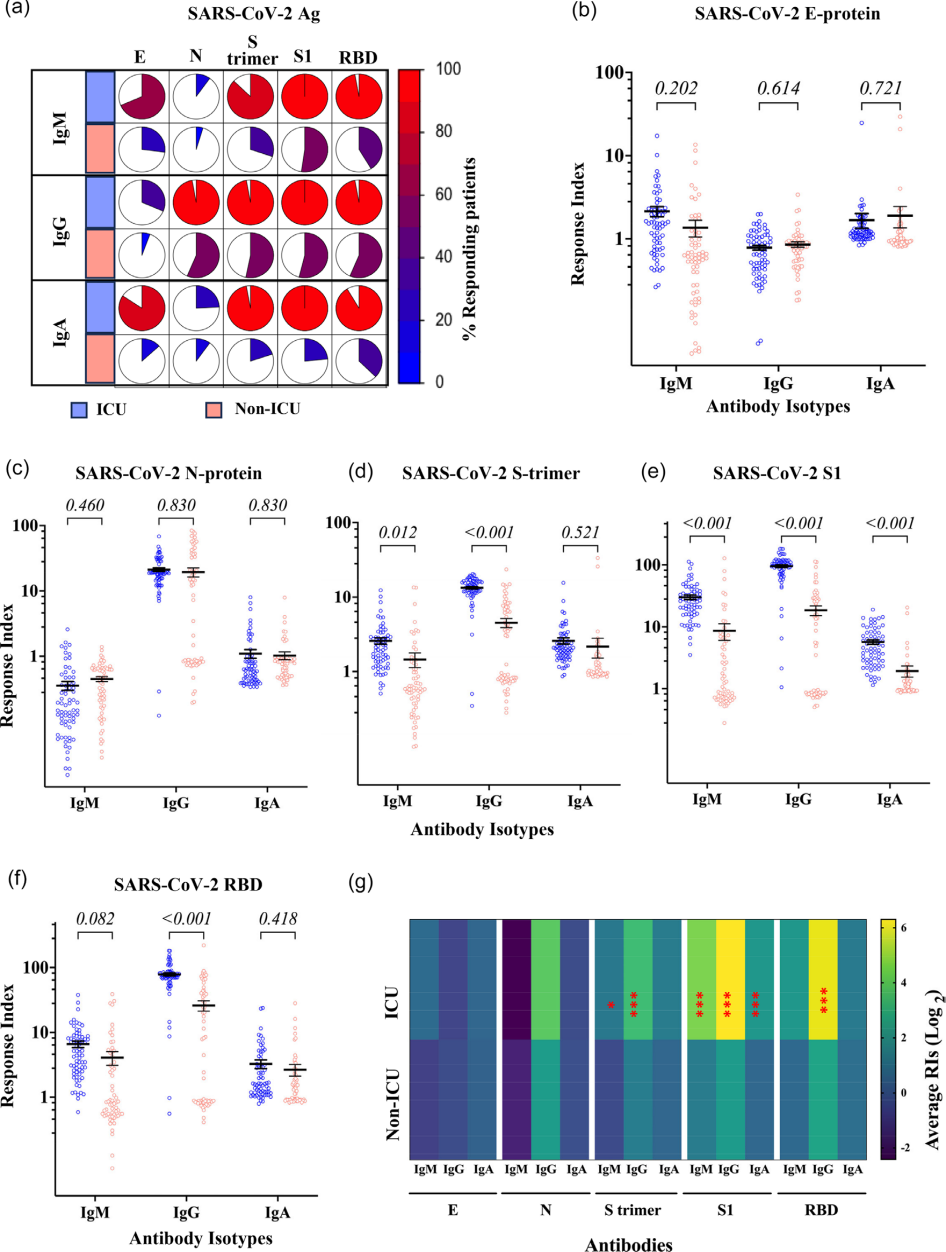
Total IgG, IgM and IgA antibody responses against SARS-CoV-2 antigens in ICU and non-ICU patients. (a) Frequency of positive Ig response in ICU and non-ICU patients. IgM, IgG and IgA RIs to SARS-CoV-2 antigens for (b) Envelope, (c) Nucleo, (d) Spike protein trimer and its fragments, (e) S1 and (f) RBD. A horizontal straight line represents the means, and the bars represent the sem. Unpaired two-tailed t-tests corrected for multiple comparisons by the Holm–Šidák correction were used to compare the ICU and non-ICU patients. (g) Heatmap analysis of the average of total IgG, IgM and IgA antibody responses against HCoVs S1 subunits. Yellow, green and blue indicate high, medium and low positivity index average values according to the scale of log2 of RIs. The significance symbols are plotted on the highest RI for each reactivity. Significance levels *: 0.01≤*P*≤ 0.05, **: 0.001≤*P*<0.01 and ***: *P*<0.001.

We analysed the sero-reactivity of antibodies to various SARS-CoV-2 antigens using an antigen flow-cytometric bead array. This analysis was performed on samples from patients hospitalized in ICU and non-ICU settings, targeting the full range of SARS-CoV-2 proteins, including the S-trimer, S1, RBD, N, M and E. The antibody levels are expressed as RI, defined by the ratio of patients’ response signals to the average response signal of non-infected patient samples collected before the COVID-19 pandemic. We first measured the frequency of patients showing a positive response, meaning having IgM, IgG or IgA RIs higher than one. We investigated the IgM, total IgG and total IgA responses to SARS-CoV-2 antigens, including E, N, S-trimer, S1 and RBD. On average, 72±16% (median±se) of ICU patients demonstrated a positive response to at least one of the measured SARS-CoV-2 antigens, while only 31±7.9% of non-ICU patients showed such a response. Positive IgG responses to Env were observed in only 31.4% of ICU patients and 5.6% of non-ICU patients. In contrast, 97.8±13.3% of ICU patients and 55±9.9% of non-ICU patients exhibited a positive IgG response against at least one of the other viral antigens: N, S-trimer, S1 and RBD (Fig. 2a).

The IgG and IgM RIs were significantly higher among ICU patients (*n*=70) than non-ICU patients (*n*=63). RIs against Envelope (E) (Fig. 2b) and Nucleocapsid (N) proteins (Fig. 2c) showed no differences between ICU and non-ICU patients (0.07<*P*<0.74 for all comparisons). When tested against the SARS-CoV-2 S-trimer, IgG RIs were 13.34±0.48 and 4.51±0.61 (*P*<0.0001) for ICU and non-ICU patients, respectively. S-trimer IgM RIs of 2.58±0.25 and 1.45±0.32 for ICU and non-ICU patients, respectively (*P*=0.012) (Fig. 2d). The increased reactivity in ICU patients was confirmed for the S protein fragment S1, with IgG RIs of 95.32±4.45 and 18.38±3.28 (*P*<0.0001) and IgM RIs of 29.98±2.59 and 8.63±2.59 (*P*<0.0001) in ICU and non-ICU patients, respectively (Fig. 2e). IgG RIs for RBD were 78.46±4.1 and 25.96±4.7 (*P*<0.0001), while IgM RIs were 6.59±0.75 and 4.08±0.97 (*P*=0.04) for ICU and non-ICU patients, respectively (Fig. 2f). At the exception of the IgA response to SARS-CoV-2 S1, which had RIs of 5.69±0.46 and 1.93±0.39 (*P*<0.0001) in ICU and non-ICU patients, respectively, IgA responses for other SARS-CoV-2 antigens showed no differences between ICU and non-ICU patients (0.42<*P*<0.73).

### Subclasses of antibody responses to SARS-CoV-2 antigens in ICU and non-ICU patients

**Fig. 3.**
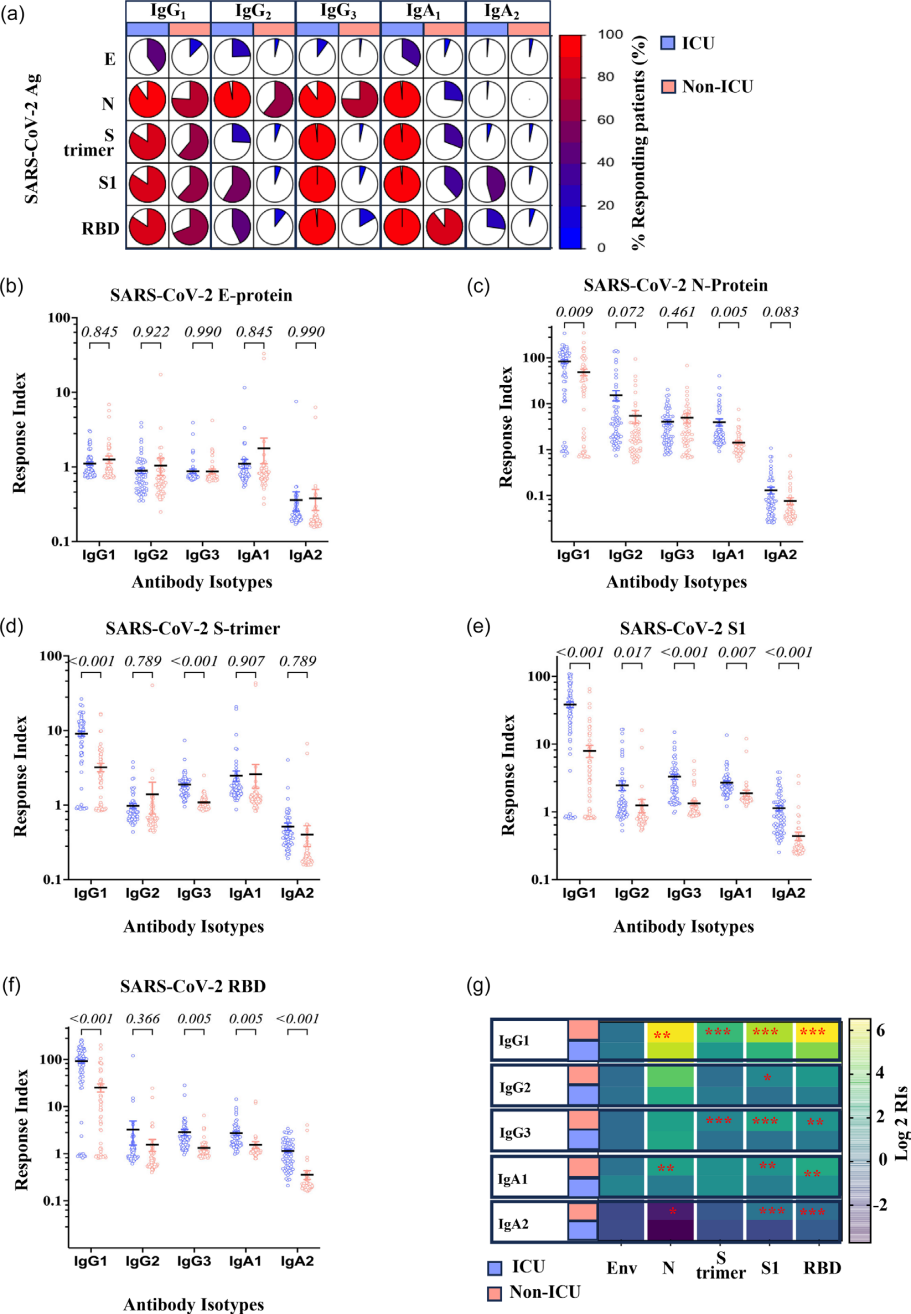
Subclass of IgG and IgA antibody responses against SARS-CoV-2 antigens in ICU and non-ICU patients. (a) Frequency of positive Ig response to SARS-CoV-2 antigens. RIs to SARS-CoV-2 antigens for (b) Envelope, (c) Nucleo, (d) Spike protein trimer and its fragments, (e) S1 and (f) RBD. A horizontal straight line represents means, and the bars represent the sem. Unpaired two-tailed t-tests corrected for multiple comparisons by the Holm–Šidák correction were used to compare the ICU and non-ICU patients. (g) Heatmap analysis of the average of total IgG, IgM and IgA antibody responses against HCoVs S1 subunits. Yellow, green and blue indicate high, medium and low positivity index average values according to the scale of log2 of RIs. Significance levels *: 0.01≤*P*≤ 0.05, **: 0.001≤*P*<0.01 and ***: *P*<0.001.

The detailed analysis of the distribution of the Ig subclasses raised against SARS-CoV-2 antigens showed a striking difference between ICU and non-ICU patients (Fig. 3a). A positive IgG1 response to SARS-CoV-2 N protein, S-trimer and its fragments S1 and RBD was observed in 85.71±1.4 and 66.94±3.4% (*P*=0.004) of ICU and non-ICU patients, respectively. IgG2 positive responses were observed in 56.1±15.3 and 20.5±13.5% (*P*=0.013) of ICU and non-ICU, respectively. IgG3 positive responses were observed in most ICU patients, at 96.8±2.3%, and only in 25.2±17.0% of non-ICU patients (*P*=0.033). The frequency of IgA1 responses also showed a significant difference between ICU and non-ICU patients, with 98.9±2.2 and 46.3±14.6% of patients responding, respectively.

The response magnitude measured by the RIs showed that ICU patients had stronger responses than non-ICU patients. However, no difference was observed for any Ig subclass in response to Envelope (Fig. 3b). IgG1 responses to N protein in ICU and non-ICU patients were 83.7±7.8 and 58.7±7.9 (*P*=2.1 10–3) (Fig. 3c); for the SARS-CoV-2 S-trimer, they were 9.04±0.7 and 3.2±0.4 (*P*=2.4 10–10) (Fig. 3d); for the S1 subunit, 39.3±3.89 and 7.33±1.52 (*P*=2.04 10–10) (Fig. 3e); 92.16±8.2 and 25.37±4.9 (*P*=4.2 10–10) for RBD (Fig. 3f), respectively. Except for IgG2 against the N protein, which had RIs of 3.5±3.9 and 1.7±1.7 (*P*=2.1 10–2) in ICU and non-ICU patients, respectively (Fig. 3c), IgG2 RIs for other SARS-CoV-2 antigens were not different between ICU and non-ICU patients (0.087<*P*<0.57). IgG3 RIs were significantly higher in ICU in comparison to non-ICU patients, who, respectively, showed RIs of 1.8±0.1 and 1.08±0.03 (*P*=8.2 10–11) for SARS-CoV-2 S-trimer, 4.66±0.5 and 1.13±0.05 (*P*=7.24 10–10) for the S1 subunit and 2.85±0.44 and 1.32±0.11 (*P*=2 10–3) for RBD. No difference was observed for IgG3 RIs to other SARS-CoV-2 antigens. The IgA1 antibody levels were significantly higher among ICU patients than non-ICU patients when tested against SARS-CoV-2 S, S1, RBD and N proteins. The IgA2 antibody levels were significantly higher among ICU patients than non-ICU patients, only in response to SARS-CoV-2 S1 and RBD. Anti-S IgG1 was the predominant IgG subtype among ICU and non-ICU SARS-CoV-2 patients, followed by IgG2 and IgG3. Anti-S IgA1 was the predominant IgA subtype among ICU and non-ICU SARS-CoV-2 patients. Overall, IgG1, IgG3 and IgA1 were the dominant Ig subclasses in ICU patients.

### Antibody responses to HCoV S1 proteins among ICU and non-ICU patients

**Fig. 4.**
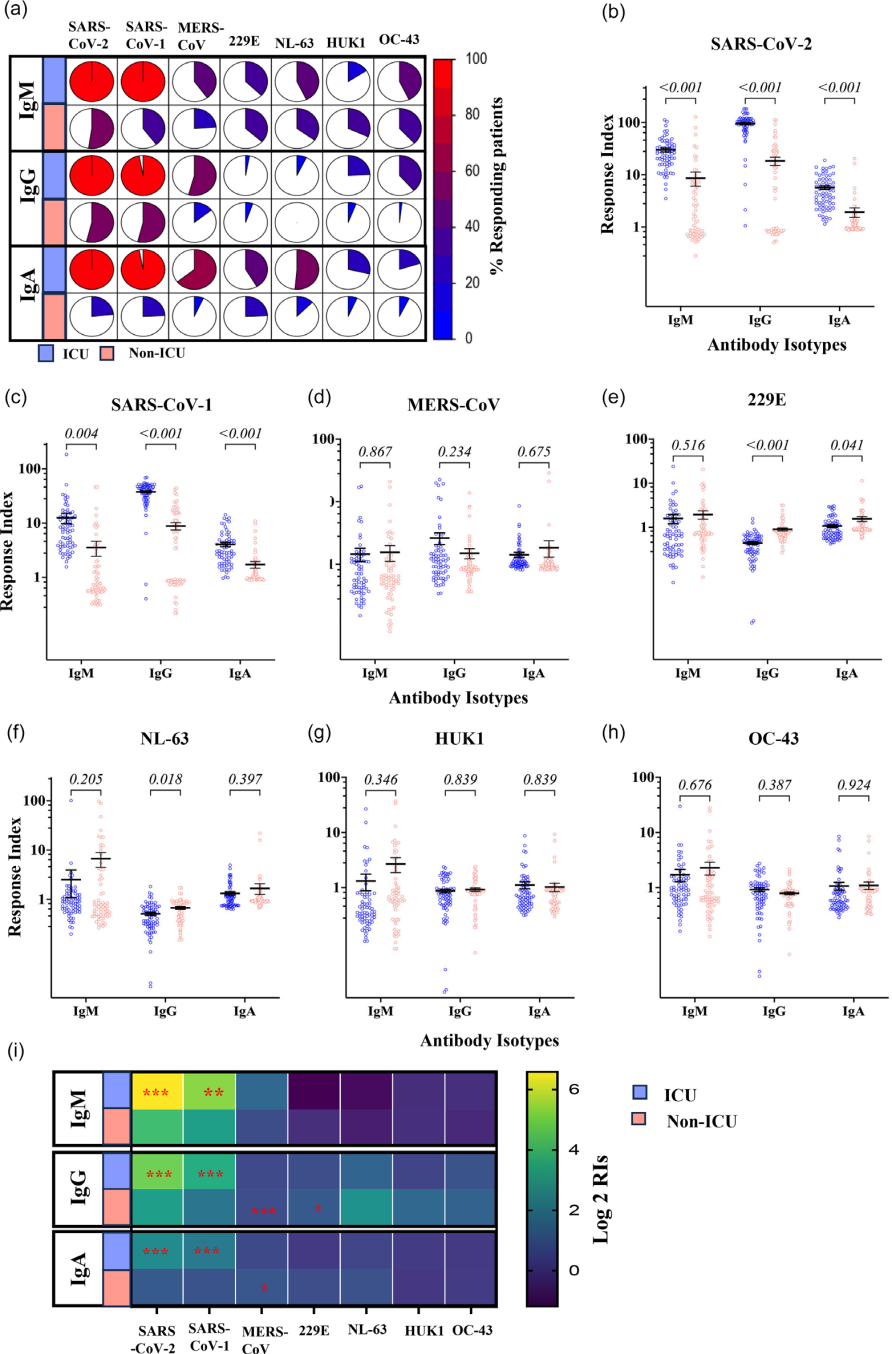
Total IgG, IgM and IgA antibody responses against HCoVs S1 proteins in ICU and non-ICU patients. (**a**) Frequency of positive Ig response. IgM, IgG and IgA RIs to HCoVs S1 proteins for (**b**) SARS-CoV-2, (**c**) SARS, (**d**) MERS, (**e**) 229E, (**f**) NL63, (**g**) HUK1 and (**h**) OC43. A horizontal straight line represents the mean, and the bars represent the sem. Unpaired two-tailed t-tests corrected for multiple comparisons by the Holm–Šidák correction were used to compare the ICU and non-ICU patients. (i) Heatmap analysis of the average of total IgG, IgM and IgA antibody responses against HCoVs S1 subunits. Yellow, green and blue indicate high, medium and low positivity index average values according to the scale of log2 of RIs. Significance levels *: 0.01≤*P*≤0.05, **: 0.001≤*P*<0.01 and ***: *P*<0.000.1.

We evaluated the extent of serological cross-reactivity with the S1 proteins of the seven HCoVs in ICU and non-ICU patient samples (Fig. 4a). SARS-CoV-2 S1 reactivity was detected in 100% of ICU patients for IgM, IgG and IgA. In contrast, these reactivities were detected in only 55.4, 60 and 23.5% of non-ICU patients. A high level of cross-reactivity was observed with SARS-CoV S, which for IgM, IgG and IgA was observed in 100, 97.1 and 97.1% of ICU patients, respectively. In non-ICU patients, as for SARS-CoV-2 S1, the reactivity to SARS-S1 was observed in fewer patients regardless of the Ig isotype. Only 39.7, 54 and 24% were positive for IgM, IgG and IgA, respectively. Although ICU patients showed greater reactivity than non-ICU patients for the two SARS viruses S1 proteins, this difference wound down for other S1 protein reactivity. In brief, 40, 54.3 and 64.3% of ICU patients had IgM, IgG and IgA recognizing MERS-CoV S1 protein. In non-ICU patients, these IgM, IgG and IgA recognizing MERS-CoV S1 were found in 45, 11 and 22.3%, respectively. IgM, IgG and IgA recognizing 229E S1 proteins were detected in 37.1, 2.9 and 41.4% of ICU patients, while the corresponding responses were observed in 36.5, 5.8 and 23.8% of non-ICU patients. In brief, 42.9, 7.1 and 51.4% of ICU patients had IgM, IgG and IgA recognizing NL63 S1 protein. In non-ICU patients, IgM, IgG and IgA recognizing this protein were found in 34.9, 0 and 7%, respectively. IgM, IgG and IgA recognizing HKU1 S1 protein were detected in 15.7, 24.3 and 28.5% of ICU patients, while the corresponding responses were observed in 37, 4.7 and 7% of non-ICU patients. Finally, we detected IgM, IgG and IgA recognizing OC43 S1 protein in 42.9, 38.6 and 20% of ICU patients. In contrast, the corresponding responses were observed in 37, 2 and 7% of non-ICU patients.

Overall, ICU patients showed extensive cross-reactive antibody responses against the SARS-CoV-2 S1 subunit. Moreover, ICU patients show slight cross-reactive IgM antibodies against NL63 and OC43 S1 subunit. The anti-S1 IgA showed slight cross-reactivity among ICU against MERS-CoV and NL63. Among non-ICU, the anti-S1 IgG showed cross-reactivity against SARS-CoV-2.

The response magnitude measured by the RIs showed that IgG, IgM and IgA RIs for SARS-CoV S1 were significantly higher in ICU patients in comparison to non-ICU patients who, respectively, showed IgG RIs of 37.61±1.66 and 8.84±1.36 (*P*<0.0001), IgM RIs of 12.47±2.63 and 3.53±1.07 (*P*=0.0035) and IgA RIs of 4.03±0.36 and 1.72±0.24 (*P*<0.0001) (Fig. 4b). IgG, IgM and IgA responses to SARS-CoV S1 were significantly higher in ICU patients in comparison to non-ICU patients who, respectively, showed IgG RIs of 37.61±1.66 and 8.84±1.36 (*P*<0.0001), IgM RIs of 12.47±2.63 and 3.53±1.07 (*P*=0.0035) and IgA RIs of 4.03±0.36 and 1.72±0.24 (*P*<0.0001) (Fig. 4c). At variance to the response to SARS-CoV-2 and SARS-CoV S1 proteins, antibody responses to 229E and NL63 S1 proteins were significantly lower in ICU patients with 229E S1 IgG RIs of 0.44±0.03 and 0.89±0.07 (*P*<0.0001) for ICU and non-ICU patients, IgA RIs of 1.07±0.07 and 1.55±0.19 (*P*=0.02) for ICU and non-ICU patients, respectively, and IgM RIs were not different (*P*=0.51) (Fig. 4e). NL63 antibody response differed only for IgG RIs which were significantly lower in ICU patients with 0.51±0.03 compared to 0.67±0.04 (*P*=0.006) for non-ICU patients (Fig. 4f). IgG, IgM and IgA RIs for MERS-CoV S1, HUK1-S1 and OC43 S1 were not significantly different between ICU and non-ICU patients (0.08<*P*<0.92) (Fig. 4d, g, h). A heat map recapitulating the RIs for the seven HCoVs S1 proteins is given (Fig. 4i).

### Detection of antibody subclass responses to HCoV S1 proteins among ICU and non-ICU patients

**Fig. 5.**
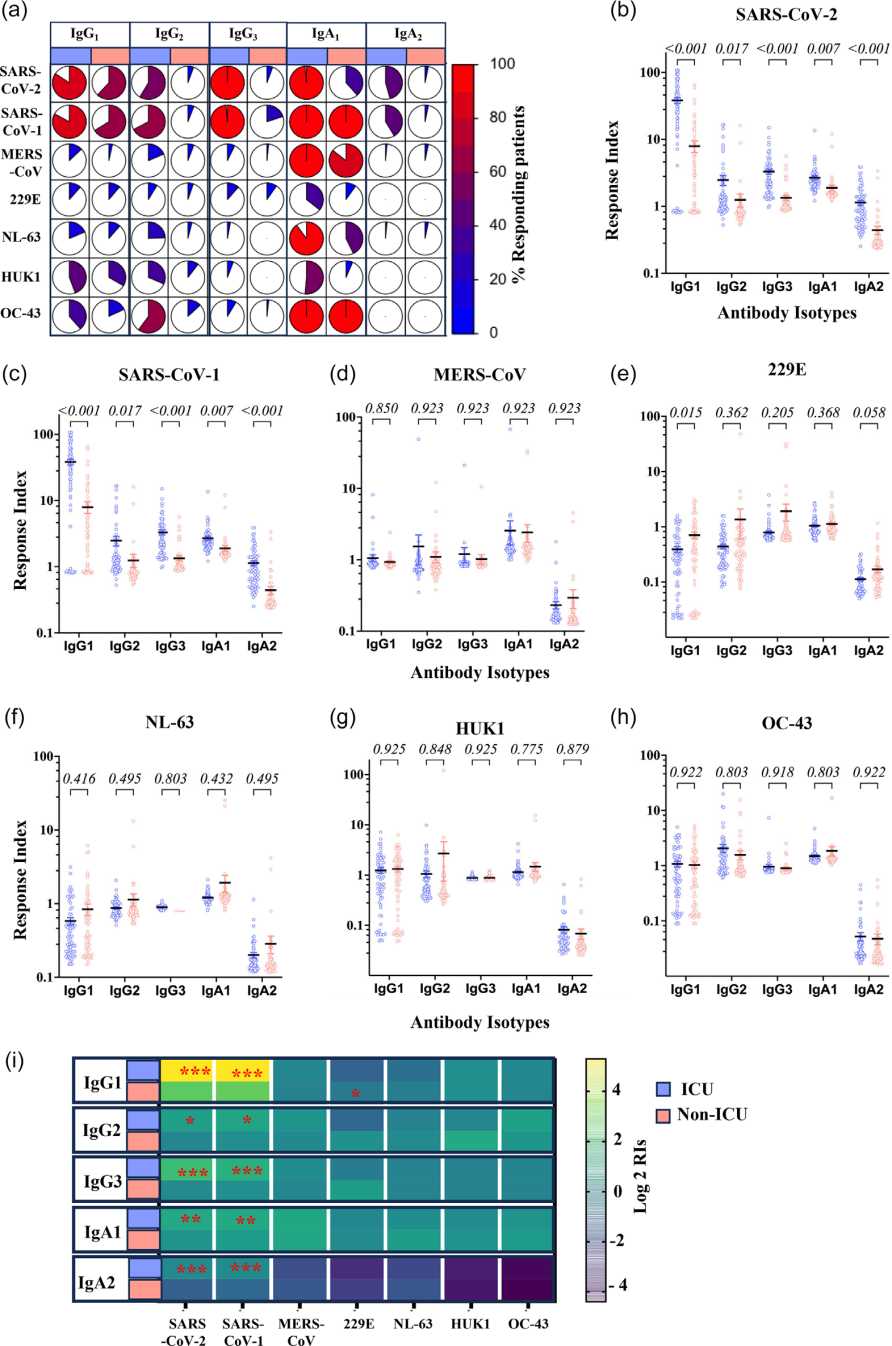
Subclass of IgG and IgA antibody responses against HCoVs S1 proteins in ICU and non-ICU patients. (a) Frequency of positive Ig subclass response to S1 proteins. RIs to HCoVs S1 proteins for (b) SARS-CoV-2, (c) SARS, (d) MERS, (e) 229E, (f) NL63, (g) HUK1 and (h) OC43. A horizontal straight line represents the mean, and the bars represent the sem. Unpaired two-tailed t-tests corrected for multiple comparisons by the Holm–Šidák correction were used to compare the ICU and non-ICU patients. (i) Heatmap analysis of the average of total IgG, IgM and IgA antibody responses against HCoVs S1 subunits. Yellow, green and blue indicate high, medium and low positivity index average values according to the scale of log2 of RIs. Significance levels *: 0.01≤*P*≤ 0.05, **: 0.001≤*P*<0.01 and ***: *P*<0.001.

The detailed analysis of the distribution of the Ig subtypes raised against HCoVs S1 protein confirmed the reactivities observed with the bulk isotypic responses reported in Fig. 5. There was a reactivity difference between ICU and non-ICU patients in recognition of SARS-CoV-2 S1 and SARS S1 (Fig. 5a). IgG1 positivity was observed in 83.6% of ICU patients, only in 64% of non-ICU patients. This difference was more pronounced for IgG2 responses, which were observed in 63% of ICU patients while reaching only 12.7% in non-ICU patients. As was the case for the response to SARS-CoV-2 antigens, IgG3 positive responses were observed in most ICU patients, with 99.3% responding and only 12.7% of non-ICU responding. The frequency of IgA1 responses also showed a difference between ICU and non-ICU patients, with 99.3 and 69.2% of patients responding, respectively.

We investigated antibody subclass responses among ICU and non-ICU patients against HCoV S1 proteins. The IgG1 RIs against SARS-CoV-2 and SARS S1 subunit were significantly higher among ICU patients than non-ICU patients. For SARS-CoV-2 S1 (Fig. 5b), they were 39.3±3.89 and 7.33±1.52 (*P*=2.04 10–10); SARS S1 RIs were 38.2±3.6 and 7.9±1.6 (*P*=2.3 10–11) (Fig. 5c). The RIs for all the other isotypes measured were significantly higher for SARS S1 reactivity in ICU patients. For IgG2, they were 2.46±0.4 and 1.24±0.27 (*P*=0.017) in ICU and non-ICU patients, respectively. IgG3 RIs in ICU and non-ICU patients were 2.46±0.4 and 1.33±0.1 (*P*=1.1 10–8), respectively. For IgA1, they were 2.68±0.2 and 1.88±0.19 (*P*=0.0034) in ICU and non-ICU patients, respectively. Finally, in ICU and non-ICU patients, IgA2 RIs were, respectively, 1.13±0.1 and 0.44±0.06 (*P*=4.9 10–8). Anti-S1 IgG1 response against MERS-CoV (Fig. 5d), NL63 (Fig. 5f), HKU1 (Fig. 5g) and OC43 (Fig. 5h) showed no significant difference between the two groups (ICU and non-ICU) (0.07<*P*<0.9). IgG1 RIs for 229E S1 were higher in non-ICU than in ICU patients, where they, respectively, were 0.71±0.1 and 0.44±0.05 (*P*=0.003) (Fig. 5e), although such levels of reactivity translate to a lack of response. The overall response levels and their significance are plotted (Fig. 5i).

### Serological response determinants of ICU

**Fig. 6.**
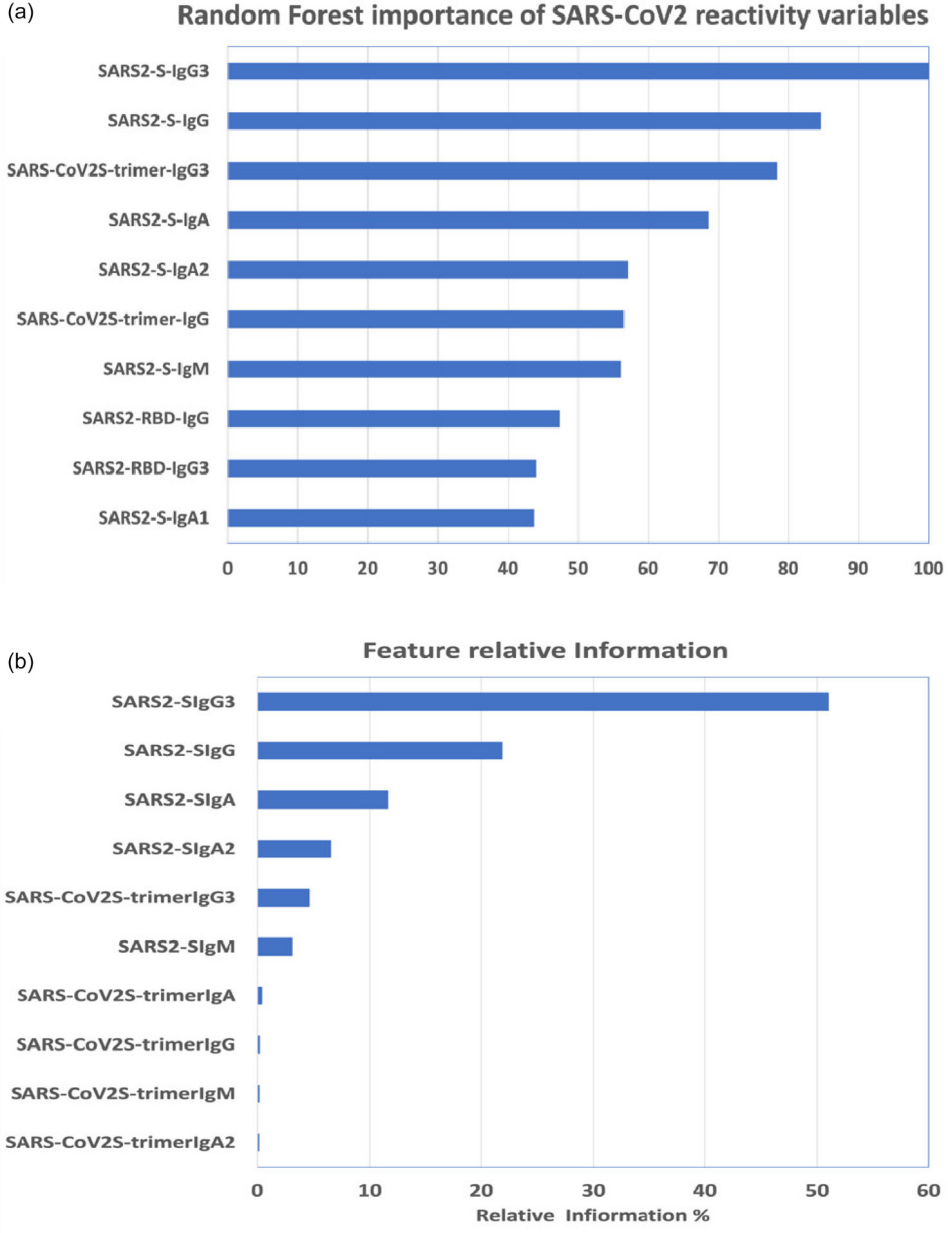
The importance of the sero-reactivity variable in determining the classification into the ICU class using (**a**) RF classification and (**b**) GBM classification.

We investigated how ICU patients differed from patients with a milder course of COVID-19. For this purpose, we built classifier models using RF or GBM algorithms. The parameters considered were age and RIs for total IgG, IgA and IgM as well as their subclasses towards all SARS-CoV-2 antigens, which constituted 41 features, which were used to build RF and GBM models with Bernoulli loss function (see method for details). Among the 41 features analysed, the RF model determined that the most important variable in determining the ICU status was IgG3 against SARS-CoV-2 S and that the top 10 variables of influence included the presence of IgG3 against S-trimer, IgG3 against RBD and IgA against SARS-CoV-2 S (Fig. 6a). The best-fitted model had an receiver operating characteristic curve (ROC) of 0.9966, a sensitivity of 0.976 and a specificity of 0.977. The best GBM model classifier defined that out of 41 predictor variables, only 9 had an influence on the classification of patients in ICU (Fig. 6b), the most influential being IgG3 against SARS-CoV-2 S and included the presence of IgG3 against S-trimer. The model had an ROC of 0.9944, a sensitivity of 0.972 and a specificity of 0.949.

To evaluate any effect of bona fide or cross-reactivity towards other HCoVs, we included total IgG, IgA, IgM and their subclasses reactive to the S1 proteins of other HCoVs, which amounted to 89 features. Modelling all 89 variables confirmed that the most significant was the presence of IgG3 against SARS-CoV-2 S and SARS-CoV-2 S-trimer. The two methods, either restricted to the sero-reactivity to SARS-CoV-2 antigens or extended to the sero-reactivity to all HCoVs, confirmed the importance of the presence of IgG3.

## Discussion

Our analysis showed that ICU patients had higher antibody responses against the SARS-CoV-2 structural proteins. A previous study reported a similar outcome [43]. In-depth analysis demonstrated higher IgG and IgM antibody responses to the SARS-CoV-2 S-trimer, S1 and RBD subunit among ICU patients compared to non-ICU patients. Similarly, several studies have reported seronegative antibody responses among asymptomatic SARS-CoV-2 patients [44,47]. Recent findings indicate elevated antibody responses to SARS-CoV-2 might stem from an impaired immune response during infection or vaccination [44,48]. A study by Hendriks *et al*. [49] found that critically ill COVID-19 patients had higher tiers and lower-affinity antibodies against the RBD, S-trimer and S2 compared to hospitalized patients [49]. The presence of low-affinity antibodies can lead to severe outcomes, as they might not effectively neutralize SARS-CoV-2, reducing immune protection and leading to increased viral load and higher inflammation. In response, the immune system increases antibody production to identify numerous targets, potentially triggering Fc-mediated immune responses, including complement activation, increased coagulation and stimulation of innate immune cells [50]. Another study analysed IgG responses to S and N proteins in hospitalized and asymptomatic COVID-19 patients. Their findings indicated that hospitalized patients had higher IgG litres for both N and S proteins, with lower avidity [46]. Low-avidity antibodies are less effective at neutralizing and clearing SARS-CoV-2 immunologically. Furthermore, recent research suggests that multiple doses of the mRNA SARS-CoV-2 vaccine may lead to increased IgG4 antibody production [51,52]. It may also inhibit the activation of both CD4+ and CD8+ T cells. Additionally, undesirable pro-inflammatory effects are linked to mRNA lipid nanoparticles [53].

Furthermore, a significant IgA response to the SARS-CoV-2 S1 subunit was recorded.

On the other hand, total IgG, IgM and IgA against SARS-CoV-2 N and E proteins showed no significant difference between ICU and non-ICU. A retrospective study conducted in China on 25 asymptomatic and 27 symptomatic SARS-CoV-2 patients showed no significant difference among IgG levels against recombinant N and S SARS-CoV-2 antigens. Furthermore, they reported significantly lower IgM responses among asymptomatic patients [54]. Another study demonstrated higher IgG and IgM antibody litres among the ICU group compared to the non-ICU group, with a significant difference for IgG litres at the 2-week post-symptom start time [55]. Furthermore, it was observed that there is a delayed specific IgM antibody response seen in COVID-19 individuals with severe disease outcomes [56]. Many factors may have contributed to different outcomes in the other studies. This includes age or pre-existing immunity (such as cross-reactive immunity from human common cold coronaviruses or innate immune subsets) [57]. It is worth noting here that samples in this study were collected at early pandemic stages; hence, the impact of vaccination and/or re-infection is limited.

Our analysis of antibody responses against the S1 subunit of HCoVs between ICU and non-ICU COVID-19 patients showed significant differences in cross-reactive immunity. Notwithstanding, our data show that other than the anti-SARS-CoV antibody response that mimicked that of SARS-CoV-2 in ICU patients, only 229E IgG, IgA and NL63 IgG were significantly higher in non-ICU patients. A cross-reactive response to SARS-CoV is expected, considering the 75% homology between the two viruses, but the homology with NL63 and 229E does not exceed 65% [22,58]. These data suggest that prior immunity against HCoVs can affect the outcome of SARS-CoV-2 infection. The S1 subunit was used to compare HCoVs due to the lack of stable S protein available commercially for all HCoVs studied. This arises because the S protein exists in two distinct forms: the pre-fusion state and the post-fusion state [59,62]. The S1 offers greater accuracy in cross-reactivity since S1 is less conserved among HCoVs than the S2 subunit [63]. The S1 harbours the key antigenic sites and the receptor-binding domain [63], making it the primary target for neutralizing antibodies. Using S1 consistently across all viruses provides a standardized platform for assessing cross-reactivity and mapping immunological relationships between coronavirus species.

Different studies have reported conflicting findings on the effect of pre-existing cross-reactive immunity to HCoV on SARS-CoV-2 infection outcomes. In an earlier study [64], a Chinese group demonstrated cross-reactivity among SARS-CoV, 229E and OC43 using an immunofluorescence (IFA) assay. Their analysis showed that 5 out of 11 and 10 out of 11 SARS-CoV patients exhibited≥4 folds increase in IgG antibody titers against 229E and OC43, respectively. Interestingly, serum samples from SARS convalescent patients had a one-way cross-reactivity with the two HCoVs. Similarly, in a recent study, cross-reactive antibody response against OC43 spike protein correlated with disease severity in COVID-19 patients [17]. This study reported higher levels of OC43 S-IgG in patients requiring mechanical ventilation. These results contrast with our observation, where we did not find significant cross-reactivity with OC43 but instead with 229E and, to a lesser extent, with NL63. In a former study [17], Guo *et al.* relied on an ELISA assay, which is less sensitive than our assay, although both studies targeted similar cohorts (severe versus non-severe COVID-19 patients during the early days of the pandemic). Still, it is particularly interesting to note a cross-reactive response with NL63 in our analysis, considering that both viruses utilize the same receptor, ACE-2. Most importantly, Wells *et al.* [65] reported elevated levels of NL63-neutralizing antibodies following exposure to SARS-CoV-2 through infection and vaccination. However, there was no substantial evidence of cross-neutralization. Hence, the cross-reactivity with NL63 S1, which harbours the RBS, as seen in our study, is not uncommon. In another study from Finland, Tamminen *et al.* [66] investigated the magnitude of HCoV antibodies in children and adults and their cross-reactivity against SARS-CoV-2. The pre-pandemic antibody cross-reactivity with SARS-CoV-2 among children and adults. Interestingly, children’s antibody levels against OC43 and 229E correlated significantly with each other and with the level of cross-reactive SARS-CoV-2 antibodies; however, these correlations were entirely lacking in adults. This could be attributed to HCoV infections in adults being controlled by memory T-cell responses, whereas in children, the immune response is more antibody-dependent. Notably, only a few studies have explored whether prior SARS-CoV-2 infection or vaccination provides protection against symptomatic HCoV infections. Bean *et al.* demonstrated prior infection with SARS-CoV-2 was associated with a low incidence of symptomatic HCoV infections, unlike vaccinated individuals [67]. Similar findings were reported by Garziano *et al.*, as they investigated the role of SARS-CoV-2 humoral immunity protecting against OC43 re-infection at either the systemic or mucosal level [68]. Their findings highlighted the protective role of SARS-CoV-2 natural infection against OC43 at systemic and mucosal levels. The contradicting outcome on the proactive role of pre-existing immunity against HCoV could be due to the reliance on binding assays rather than assays that measure antibody functions such as neutralization, antibody-sependent cellular cytotoxicity (ADCC), antibody-dependent phagocytosis of cells (ADCP) and others. Hence, correlating cross-reactive antibody responses with severe disease outcomes couldn’t be made. Thus, it remains unclear whether exposure to one virus would increase protection against the other.

On the other hand, it is essential to determine the epitopes targeted by cross-reactive antibodies, as that would help in structure-based vaccine design. Several studies reported that the S2 subdomain of the S protein is the main target for cross-reactivity [57,69, 70], considering the high degree of similarity in this region. We did not incorporate S2 in our assay, as we could not find a well-characterized S2 antigen, which is also a drawback of other reports that did not structurally define the S2 antigen in their analysis [57,69, 70].

Immunoglobulin subclass analysis revealed a uniform pattern: IgG1 against S antigens (whole S, S1 and, to a lesser extent, RBD) was significantly higher in ICU patients than in non-ICU patients. Interestingly, the IgG3 but not IgG2 response against S antigens was elevated in ICU patients. Similar observations were made regarding reactivity to SARS-CoV but not to other HCoV. IgG1 is the most prevalent IgG subtype in human serum [71] and is usually elevated after viral infection. It usually binds viral pathogens by binding to soluble and membrane proteins [71].

On the other hand, the involvement of IgG3 in the pathogenesis of acute respiratory distress syndrome (ARDS) in COVID-19 patients has been recorded [72,74]. In a recent study, Iles *et al.* [72] used maldi-tof mass spectrometry (MALDI-TOF MS) to analyse antibody response against S and N antigens. Differential analysis of the spectral signatures demonstrated that the predominant humoral immune response to the nucleocapsid was IgG3, while the spike protein was IgG1. In our study, IgG1 was dominant over IgG3 to both S and N antigens and was significantly higher in ICU patients. Generally speaking, IgG3 Abs are potent mediators of effector functions, including neutralization, enhanced ADCC, complement activation and opsonophagocytosis, compared with other IgG subclasses [75]. IgG1 and IgG3 have been reported to play a role in enhanced disease illness [76] and were elevated in ICU patients. Higher levels of IgG1 and IgG3 were found among SARS-CoV-2 patients in other studies [77]. A study by Luo *et al.* showed that elevated levels of anti-N IgG1 and IgG3 are associated with disease severity [77]. Another study showed an increased level of anti-RBD IgG1 and IgG3 in the ICU compared to non-ICU SARS-CoV-2 patients, consistent with our findings [78]. Further functional characterization of IgG and IgG3 antibodies is needed to understand their role in COVID-19 disease severity.

We have utilized a novel antigen bead array to assess anti-HCoV responses among ICU and non-ICU patients. The aim was to see whether specific antibody responses against HCoV might correlate with the clinical outcome of SARS-CoV-2-infected patients. Understanding the different antibody responses between ICU and non-ICU patients is crucial to answering emerging questions, such as the possibility of pre-existing immunity against SARS-CoV-2 resulting in different magnitudes in disease severity.

Antigen arrays are powerful tools for multiplex detection of antibody landscapes that can provide in-depth analysis of cross-reactive responses [79]. These high-throughput screening assays could replace well-known indirect tests such as ELISA. While many single-target assays demonstrate excellent sensitivity and specificity, the assay’s performance can be significantly impacted when the testing is performed at a low seroprevalence profile, and it hardly differentiates cross-reactive antibodies against other pathogens [80]. Much research has been done to assess antibody litres and temporal profiles in COVID-19 patients with varying illness severity [81,83]. Here, we present an in-house antigen bead assay that measures antibody responses against the seven HCoVs that infect humans. The assay measures the antibody litres, the subclasses to five SARS-CoV-2 antigens (S, S1, RBD, N and E) and the HCoV S1 proteins. The assay was used to profile antibody responses in severe (ICU) and non-severe COVID-19 patients.

## Conclusion

In conclusion, our findings emphasized the role of anti-S IgG3 of SARS-CoV-2 as a biomarker of COVID-19 disease severity. This was supported by recent evidence indicating the involvement of IgG3 in the pathogenesis of ARDS among COVID-19 patients [72,74]. In addition, our findings illustrate the antibody responses among SARS-CoV-2 ICU and non-ICU patients utilizing the antigen bead array. Antigen bead array is more sensitive, enabling the detection of low levels of antibodies present in the sample and, hence, accurate results [84]. Moreover, it is a faster method that can simultaneously detect multiple antibodies in the sample. Thus, exploring the antibody responses against different HCoVs well-characterized antigens can be beneficial. It can be implemented to study the cross-reactive antibodies against other viruses, as there has been substantial debate over the potential role of cross-protective immunity to SARS-CoV-2 infection due to previous seasonal HCoV infection [16,85]. One of the limitations of our study is that we haven’t assessed the role of cross-reactive T-cell response against HCoVs. Several published reports have identified the cross-reactive T-cell between SARS-CoV-2 and HCoVs in unexposed individuals to SARS-CoV-2 [86,89]. Cross-reactive T-cell plays a significant role in controlling SARS-CoV-2 immunity, potentially contributing to severe disease outcomes and enhancing vaccine responses. Further research is needed to understand their implications for SARS-CoV-2 disease severity fully. A deep understanding of coronavirus cross-protection requires adequately designed research, such as longitudinal cohorts or experimental infection studies, ideally with an investigation of both humoral and cellular immunity. Notably, we find evidence for significant cross-reactivity between antibodies to SARS-CoV-2 and SARS-CoV-1 but no substantial evidence for cross-protective immunity to SARS-CoV-2 infection due to previous seasonal HCoV infection.

## Abbreviations

ACE-2: angiotensin-converting enzyme 2
ADCC: antibody-dependent cell-mediated cytotoxicity
ADCP: antibody-dependent cellular phagocytosis
ARDS: acute respiratory distress syndrome
GBM: gradient-boosted machine
HCoVs: human coronaviruses
IFA: immunofluorescence assay
IQR: interquartile range
MALDI-TOF: matrix-assisted laser desorption ionization- time of flight
MERS-CoV: middle east respiratory syndrome coronavirus
MFI: mean fluorescence intensity
PE: phycoerythrin
RBD: receptor binding domain
RF: random forest
RI: response index
ROC: receiver operating characteristic
RT-PCR: real time polymerase chain reaction
SARS-CoV: severe acute respiratory syndrome coronavirus
WHO: World Health Organization

## References

[1] Bhat EA, Khan J, Sajjad N, Ali A, Aldakeel FM (2021). SARS-CoV-2: Insight in genome structure, pathogenesis and viral receptor binding analysis - An updated review. Int Immunopharmacol.

[2] Kulshrestha S, Verma YK (2024). Stem Cells.

[3] Andersen KG, Rambaut A, Lipkin WI, Holmes EC, Garry RF (2020). The proximal origin of SARS-CoV-2. Nat Med.

[4] Çelik I, Öztürk R (2021). From asymptomatic to critical illness: decoding various clinical stages of COVID-19. Turk J Med Sci.

[5] Gao Z, Xu Y, Sun C, Wang X, Guo Y (2021). A systematic review of asymptomatic infections with COVID-19. J Microbiol Immunol Infect.

[6] Wratil PR, Schmacke NA, Karakoc B, Dulovic A, Junker D (2021). Evidence for increased SARS-CoV-2 susceptibility and COVID-19 severity related to pre-existing immunity to seasonal coronaviruses. Cell Rep.

[7] Sette A, Crotty S (2020). Pre-existing immunity to SARS-CoV-2: the knowns and unknowns. Nat Rev Immunol.

[8] Mantus G, Nyhoff LE, Edara V-V, Zarnitsyna VI, Ciric CR (2022). Pre-existing SARS-CoV-2 immunity influences potency, breadth, and durability of the humoral response to SARS-CoV-2 vaccination. Cell Rep Med.

[9] Denning DW, Kilcoyne A, Ucer C (2020). Non-infectious status indicated by detectable IgG antibody to SARS-CoV-2. Br Dent J.

[10] Sterlin D, Mathian A, Miyara M, Mohr A, Anna F (2021). IgA dominates the early neutralizing antibody response to SARS-CoV-2. Sci Transl Med.

[11] Yamayoshi S, Yasuhara A, Ito M, Akasaka O, Nakamura M (2021). Antibody titers against SARS-CoV-2 decline, but do not disappear for several months. EClinicalMedicine.

[12] Tarkowski M, de Jager W, Schiuma M, Covizzi A, Lai A (2021). Anti-SARS-CoV-2 immunoglobulin isotypes, and neutralization activity against viral variants, according to BNT162b2-vaccination and infection history. Front Immunol.

[13] Du L, Yang Y, Zhang X (2021). Neutralizing antibodies for the prevention and treatment of COVID-19. *Cell Mol Immunol*.

[14] Tso FY, Lidenge SJ, Peña PB, Clegg AA, Ngowi JR (2021). High prevalence of pre-existing serological cross-reactivity against severe acute respiratory syndrome coronavirus-2 (SARS-CoV-2) in sub-Saharan Africa. Int J Infect Dis.

[15] Shiakolas AR, Kramer KJ, Wrapp D, Richardson SI, Schäfer A (2021). Cross-reactive coronavirus antibodies with diverse epitope specificities and Fc effector functions. *Cell Rep Med*.

[16] Anderson EM, Goodwin EC, Verma A, Arevalo CP, Bolton MJ (2021). Seasonal human coronavirus antibodies are boosted upon SARS-CoV-2 infection but not associated with protection. Cell.

[17] Guo L, Wang Y, Kang L, Hu Y, Wang L (2021). Cross-reactive antibody against human coronavirus OC43 spike protein correlates with disease severity in COVID-19 patients: a retrospective study. Emerg Microbes Infect.

[18] Hicks J, Klumpp-Thomas C, Kalish H, Shunmugavel A, Mehalko J (2021). Serologic cross-reactivity of SARS-CoV-2 with endemic and seasonal Betacoronaviruses. J Clin Immunol.

[19] Zheng Z, Monteil VM, Maurer-Stroh S, Yew CW, Leong C (2020). Monoclonal antibodies for the S2 subunit of spike of SARS-CoV-1 cross-react with the newly-emerged SARS-CoV-2. Euro Surveill.

[20] AlKhalifah JM, Seddiq W, Alshehri MA, Alhetheel A, Albarrag A (2023). Impact of MERS-CoV and SARS-CoV-2 viral infection on immunoglobulin-IgG cross-reactivity. Vaccines.

[21] Hatmal MM, Alshaer W, Al-Hatamleh MAI, Hatmal M, Smadi O (2020). Comprehensive structural and molecular comparison of spike proteins of SARS-CoV-2, SARS-CoV and MERS-CoV, and their interactions with ACE2. Cells.

[22] Gorkhali R, Koirala P, Rijal S, Mainali A, Baral A (2021). Structure and function of major SARS-CoV-2 and SARS-CoV proteins. Bioinform Biol Insights.

[23] Goyal R, Gautam RK, Chopra H, Dubey AK, Singla RK (2022). Comparative highlights on MERS-CoV, SARS-CoV-1, SARS-CoV-2, and NEO-CoV. EXCLI J.

[24] Ng KW, Faulkner N, Cornish GH, Rosa A, Harvey R (2020). Preexisting and de novo humoral immunity to SARS-CoV-2 in humans. Science.

[25] Grant MD, Bentley K, Fielding CA, Hatfield KM, Ings DP (2023). Hybrid immunity elicits potent cross-variant ADCC against SARS-CoV-2 through a combination of anti-S1 and S2 antibodies. Immunology.

[26] Chouchane L, Grivel J-C, Farag EABA, Pavlovski I, Maacha S (2021). Dromedary camels as a natural source of neutralizing nanobodies against SARS-CoV-2. JCI Insight.

[27] Engelbrecht F, Madhi S, Scholes R (2021). Pandemic-stage propagation dynamics in South Africa suggest pre-existing cross-reactive protection against severe Covid-19. In Rev.

[28] Murray SM, Ansari AM, Frater J, Klenerman P, Dunachie S (2023). The impact of pre-existing cross-reactive immunity on SARS-CoV-2 infection and vaccine responses. Nat Rev Immunol.

[29] Kober C, Manni S, Wolff S, Barnes T, Mukherjee S (2022). IgG3 and IgM identified as key to SARS-CoV-2 neutralization in convalescent plasma pools. PLoS One.

[30] Sun L, Kallolimath S, Palt R, Stiasny K, Mayrhofer P (2021). Increased *in vitro* neutralizing activity of SARS-CoV-2 IgA1 dimers compared to monomers and IgG. Proc Natl Acad Sci USA.

[31] Noval MG, Kaczmarek ME, Koide A, Rodriguez-Rodriguez BA, Louie P (2021). Antibody isotype diversity against SARS-CoV-2 is associated with differential serum neutralization capacities. Sci Rep.

[32] Wang H, Yan D, Li Y, Gong Y, Mai Y (2022). Clinical and antibody characteristics reveal diverse signatures of severe and non-severe SARS-CoV-2 patients. Infect Dis Poverty.

[33] Gudbjartsson DF, Norddahl GL, Melsted P, Gunnarsdottir K, Holm H (2020). Humoral immune response to SARS-CoV-2 in Iceland. N Engl J Med.

[34] Rubio-Rivas M, Mora-Luján JM, Formiga F, Arévalo-Cañas C, Lebrón Ramos JM (2022). WHO ordinal scale and inflammation risk categories in COVID-19. Comparative study of the severity scales. J Gen Intern Med.

[35] Benslimane FM, Al Khatib HA, Al-Jamal O, Albatesh D, Boughattas S (2021). One year of SARS-CoV-2: genomic characterization of COVID-19 outbreak in Qatar. Front Cell Infect Microbiol.

[36] Wilson R, Kovacs D, Crosby M, Ho A (2024). Global epidemiology and seasonality of human seasonal coronaviruses: a systematic review. Open Forum Infect Dis.

[37] Biancotto A, Brichacek B, Chen SS, Fitzgerald W, Lisco A (2009). A highly sensitive and dynamic immunofluorescent cytometric bead assay for the detection of HIV-1 p24. J Virol Methods.

[38] Šidák Z (1967). Rectangular confidence regions for the means of multivariate normal distributions. J Am Stat Assoc.

[39] Holm S (1979). A simple sequentially rejective multiple test procedure. Scand Statist.

[40] Sokollik C, Pahud de Mortanges A, Leichtle AB, Juillerat P, Horn MP (2023). Machine learning in antibody diagnostics for inflammatory bowel disease subtype classification. Diagnostics.

[41] Kuhn M (2013). Applied Predictive Modeling.

[42] Kuhn M (2008). Building predictive models in R using the caret package. J Stat Softw.

[43] Sun B, Feng Y, Mo X, Zheng P, Wang Q (2020). Kinetics of SARS-CoV-2 specific IgM and IgG responses in COVID-19 patients. Emerg Microbes Infect.

[44] Liu L, To KK-W, Chan K-H, Wong Y-C, Zhou R (2020). High neutralizing antibody titer in intensive care unit patients with COVID-19. Emerg Microbes Infect.

[45] Tamizuddin S, Cham J, Ghiasi Y, Borroto L, Cao C (2022). Hospitalization requiring intensive care unit due to SARS-CoV-2 infection correlated with IgM depression and IgG elevation. Future Sci OA.

[46] Hajilooi M, Keramat F, Moazenian A, Rastegari-Pouyani M, Solgi G (2023). The quantity and quality of anti-SARS-CoV-2 antibodies show contrariwise association with COVID-19 severity: lessons learned from IgG avidity. Med Microbiol Immunol.

[47] Liao B, Chen Z, Zheng P, Li L, Zhuo J (2021). Detection of anti-SARS-CoV-2-S2 IgG is more sensitive than anti-RBD IgG in identifying asymptomatic COVID-19 patients. Front Immunol.

[48] Ibrahim EH, Alshahrani MY, Ghramh HA, Kilany M (2021). Antibody profile in symptomatic/asymptomatic severe acute respiratory syndrome coronavirus 2 (SARS-CoV-2) infected Saudi persons. Saudi J Biol Sci.

[49] Hendriks J, Schasfoort R, Koerselman M, Dannenberg M, Cornet AD (2022). High titers of low affinity antibodies in COVID-19 patients are associated with disease severity. Front Immunol.

[50] Anand SP, Finzi A (2019). Understudied factors influencing Fc-mediated immune responses against viral infections. Vaccines.

[51] Gazit S, Shlezinger R, Perez G, Lotan R, Peretz A (2022). Severe acute respiratory syndrome coronavirus 2 (SARS-CoV-2) naturally acquired immunity versus vaccine-induced immunity, reinfections versus breakthrough infections: a retrospective cohort study. Clin Infect Dis.

[52] Shrestha NK, Burke PC, Nowacki AS, Simon JF, Hagen A (2023). Effectiveness of the coronavirus disease 2019 bivalent vaccine. Open Forum Infect Dis.

[53] Trougakos IP, Terpos E, Alexopoulos H, Politou M, Paraskevis D (2022). Adverse effects of COVID-19 mRNA vaccines: the spike hypothesis. Trends Mol Med.

[54] Han H, Xu Z, Cheng X, Zhong Y, Yuan L (2020). Descriptive, retrospective study of the clinical characteristics of asymptomatic COVID-19 patients. mSphere.

[55] To KK-W, Tsang OT-Y, Leung W-S, Tam AR, Wu T-C (2020). Temporal profiles of viral load in posterior oropharyngeal saliva samples and serum antibody responses during infection by SARS-CoV-2: an observational cohort study. Lancet Infect Dis.

[56] Shen L, Wang C, Zhao J, Tang X, Shen Y (2020). Delayed specific IgM antibody responses observed among COVID-19 patients with severe progression. Emerg Microbes Infect.

[57] Grobben M, van der Straten K, Brouwer PJ, Brinkkemper M, Maisonnasse P (2021). Cross-reactive antibodies after SARS-CoV-2 infection and vaccination. elife.

[58] Bačenková D, Trebuňová M, Špakovská T, Schnitzer M, Bednarčíková L (2021). Comparison of selected characteristics of SARS-CoV-2, SARS CoV, and HCoV-NL63. Appl Sci.

[59] Zhang K, Li S, Pintilie G, Chmielewski D, Schmid MF (2020). A 3.4-Å cryo-electron microscopy structure of the human coronavirus spike trimer computationally derived from vitrified NL63 virus particles. *QRB Discov*.

[60] Yu J, Qiao S, Guo R, Wang X (2020). Cryo-EM structures of HKU2 and SADS-CoV spike glycoproteins provide insights into coronavirus evolution. Nat Commun.

[61] Li Z, Tomlinson AC, Wong AH, Zhou D, Desforges M (2019). The human coronavirus HCoV-229E S-protein structure and receptor binding. elife.

[62] Huang C-Y, Hsu Y-L, Chiang W-L, Hou M-H (2009). Elucidation of the stability and functional regions of the human coronavirus OC43 nucleocapsid protein. Protein Sci.

[63] Huang Y, Yang C, Xu X, Xu W, Liu S (2020). Structural and functional properties of SARS-CoV-2 spike protein: potential antivirus drug development for COVID-19. Acta Pharmacol Sin.

[64] Che X-Y, Qiu L-W, Liao Z-Y, Wang Y, Wen K (2005). Antigenic cross-reactivity between severe acute respiratory syndrome-associated coronavirus and human coronaviruses 229E and OC43. J Infect Dis.

[65] Wells DA, Cantoni D, Mayora-Neto M, Genova CD, Sampson A (2022). Human seasonal coronavirus neutralization and COVID-19 severity. J Med Virol.

[66] Tamminen K, Salminen M, Blazevic V (2021). Seroprevalence and SARS-CoV-2 cross-reactivity of endemic coronavirus OC43 and 229E antibodies in Finnish children and adults. Clin Immunol.

[67] Bean DJ, Monroe J, Liang YM, Borberg E, Senussi Y (2024). Heterotypic immunity from prior SARS-CoV-2 infection but not COVID-19 vaccination associates with lower endemic coronavirus incidence. Sci Transl Med.

[68] Garziano M, Cano Fiestas M, Vanetti C, Strizzi S, Murno ML (2024). SARS-CoV-2 natural infection, but not vaccine-induced immunity, elicits cross-reactive immunity to OC43. Heliyon.

[69] Shah P, Canziani GA, Carter EP, Chaiken I (2021). The case for S2: the potential benefits of the S2 subunit of the SARS-CoV-2 spike protein as an immunogen in fighting the COVID-19 pandemic. Front Immunol.

[70] Guo L, Lin S, Chen Z, Cao Y, He B (2023). Targetable elements in SARS-CoV-2 S2 subunit for the design of pan-coronavirus fusion inhibitors and vaccines. Signal Transduct Target Ther.

[71] Vidarsson G, Dekkers G, Rispens T (2014). IgG subclasses and allotypes: from structure to effector functions. Front Immunol.

[72] Iles JK, Zmuidinaite R, Sadee C, Gardiner A, Lacey J (2022). Determination of IgG1 and IgG3 SARS-CoV-2 spike protein and nucleocapsid binding—Who is binding who and why?. Int J Mol Sci.

[73] Cervia C, Zurbuchen Y, Taeschler P, Ballouz T, Menges D (2022). Immunoglobulin signature predicts risk of post-acute COVID-19 syndrome. Nat Commun.

[74] Yates JL, Ehrbar DJ, Hunt DT, Girardin RC, Dupuis A (2020). Serological analysis reveals an imbalanced IgG subclass composition associated with COVID-19 disease severity. Infect Dis.

[75] Damelang T, Rogerson SJ, Kent SJ, Chung AW (2019). Role of IgG3 in infectious diseases. Trends Immunol.

[76] Espino AM, Armina-Rodriguez A, Alvarez L, Ocasio-Malavé C, Ramos-Nieves R (2024). The anti-SARS-CoV-2 IgG1 and IgG3 antibody isotypes with limited neutralizing capacity against Omicron elicited in a Latin population: a switch toward IgG4 after multiple doses with the mRNA Pfizer-BioNTech vaccine. Viruses.

[77] Luo H, Jia T, Chen J, Zeng S, Qiu Z (2021). The characterization of disease severity-associated IgG subclasses response in COVID-19 patients. Front Immunol.

[78] Zhang W, Chua BY, Selva KJ, Kedzierski L, Ashhurst TM (2022). SARS-CoV-2 infection results in immune responses in the respiratory tract and peripheral blood that suggest mechanisms of disease severity. Nat Commun.

[79] Ayoglu B, Szarka E, Huber K, Orosz A, Babos F (2014). Bead arrays for antibody and complement profiling reveal joint contribution of antibody isotypes to C3 deposition. PLoS One.

[80] Mitchell KF, Carlson CM, Nace D, Wakeman BS, Drobeniuc J (2022). Evaluation of a multiplex bead assay against single-target assays for detection of IgG antibodies to SARS-CoV-2. Microbiol Spectr.

[81] Liu L, Chen H-G, Li Y, Li H, Li J (2021). Temporal profiles of antibody responses, cytokines, and survival of COVID-19 patients: a retrospective cohort. Engineering.

[82] Long Q-X, Liu B-Z, Deng H-J, Wu G-C, Deng K (2020). Antibody responses to SARS-CoV-2 in patients with COVID-19. Nat Med.

[83] Yongchen Z, Shen H, Wang X, Shi X, Li Y (2020). Different longitudinal patterns of nucleic acid and serology testing results based on disease severity of COVID-19 patients. Emerg Microbes Infect.

[84] Reed EF, Rao P, Zhang Z, Gebel H, Bray RA (2013). Comprehensive assessment and standardization of solid phase multiplex-bead arrays for the detection of antibodies to HLA. Am J Transplant.

[85] Dobaño C, Santano R, Jiménez A, Vidal M, Chi J (2021). Immunogenicity and crossreactivity of antibodies to the nucleocapsid protein of SARS-CoV-2: utility and limitations in seroprevalence and immunity studies. Transl Res.

[86] Grifoni A, Weiskopf D, Ramirez SI, Mateus J, Dan JM (2020). Targets of T cell responses to SARS-CoV-2 coronavirus in humans with COVID-19 disease and unexposed individuals. Cell.

[87] Le Bert N, Tan AT, Kunasegaran K, Tham CYL, Hafezi M (2020). SARS-CoV-2-specific T cell immunity in cases of COVID-19 and SARS, and uninfected controls. Nature.

[88] Braun J, Loyal L, Frentsch M, Wendisch D, Georg P (2020). SARS-CoV-2-reactive T cells in healthy donors and patients with COVID-19. Nature.

[89] Sałkowska A, Karwaciak I, Karaś K, Dastych J, Ratajewski M (2020). SARS-CoV-2 proteins induce ifng in Th1 lymphocytes generated from CD4+ cells from healthy, unexposed polish donors. Vaccines.

